# Supplementary material for: Coupled Electrostatic and Hydrophobic Destabilisation of the Gelsolin-Actin Complex Enables Facile Detection of Ovarian Cancer Biomarker Lysophosphatidic Acid
Source: Biomolecules. 2023 Sep 21;13(9):1426. doi: 10.3390/biom13091426 (PMC10527313; doi:10.3390/biom13091426)
Supplement: Supplementary file 1 [file biomolecules-13-01426-s001.zip › biomolecules-2564575-supplementary.pdf]

Supplementary Information

Coupled Electrostatic and Hydrophobic Destabilisation of the Gelsolin-Actin Complex Enables Facile Detection of Ovarian Cancer Biomarker Lysophosphatidic Acid

Katharina Davoudian [1], Shayon Bhattacharya [2,3], Damien Thompson [2,3,*] and Michael Thompson [1,*]

[1] Department of Chemistry, University of Toronto, 80 St. George Street, Toronto, ON M5S 3H6, Canada; k.davoudian@mail.utoronto.ca
[2] SSPC, The Science Foundation Ireland Research Centre for Pharmaceuticals, V94 T9PX Limerick, Ireland; shayon.bhattacharya@ul.ie
[3] Department of Physics, Bernal Institute, University of Limerick, V94 T9PX Limerick, Ireland
* Correspondence: damien.thompson@ul.ie (D.T.); m.thompson@utoronto.ca (M.T.)

Supplementary Notes

S1. Lysophosphatidic Acid Ionisation

Before docking of LPA to the gelsolin(1-3)-actin complex, the protonation state of LPA at pH 7.4 was calculated using the Schrödinger Maestro package [38]. It was predicted that LPA has two deprotonated oxygens in the phosphate head group (PO^{2-}). Therefore, LPA was modelled to have a net -2e negative charge under physiological pH.

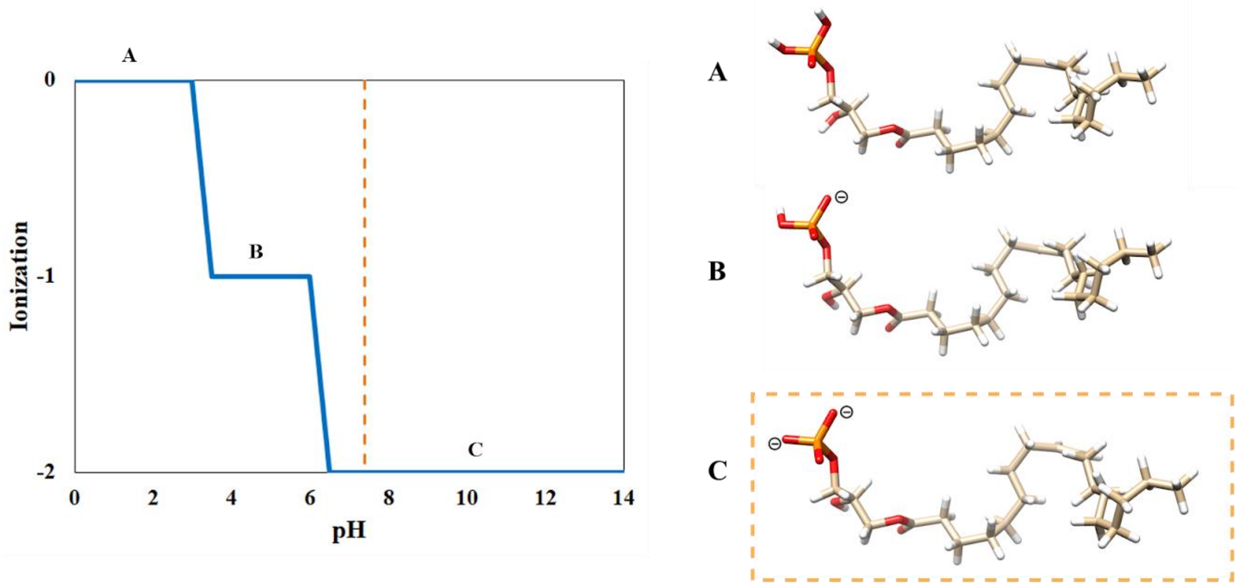

Figure S1. Protonation state of LPA at physiological pH 7.4 (dashed orange line). The left plot shows that LPA is predicted to have -2e charge. Structures of **(A)** Neutral LPA, **(B)** -1e charged LPA with one deprotonated oxygen of the phosphate headgroup, and **(C)** -2e charged LPA with two deprotonated oxygens of the phosphate headgroup.

S2-3. Hydrogen Bonds between Gelsolin(1-3) and Actin

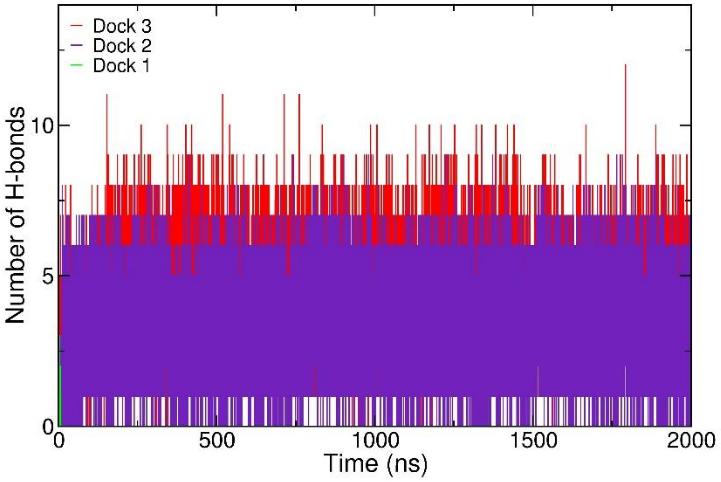

Figure S2. The number of H-bonds between LPA and the gelsolin(1-3)-actin complex as a function of simulation timeline. Dock 1 is green (lower left), as LPA leaves the binding pocket and loses H-bonds with the protein ~6 ns.

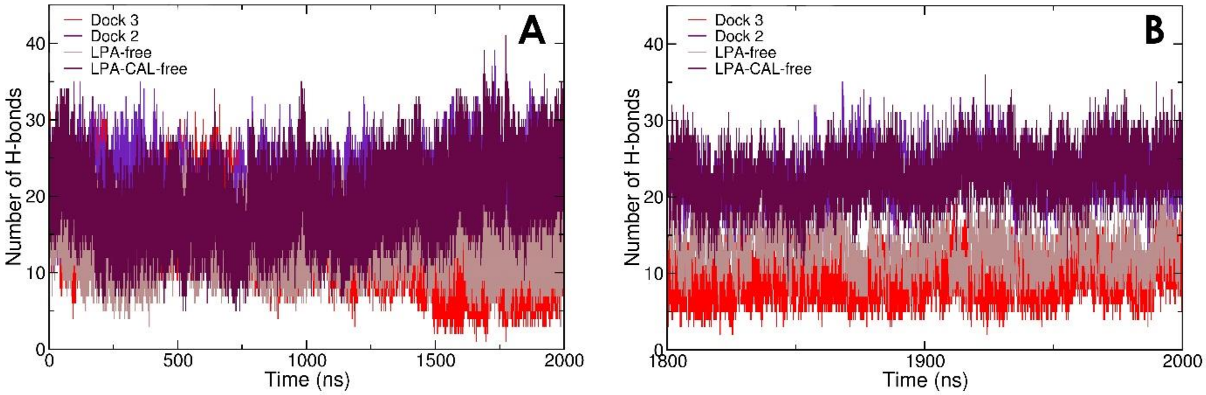

Figure S3. Number of hydrogen bonds between gelsolin 1-3 and actin across **(A)** the entire simulation time and **(B)** the last 200ns.

S4. Hydrogen Bonds and Interaction Energy between Gelsolin(1-3) and Actin in Dock 1

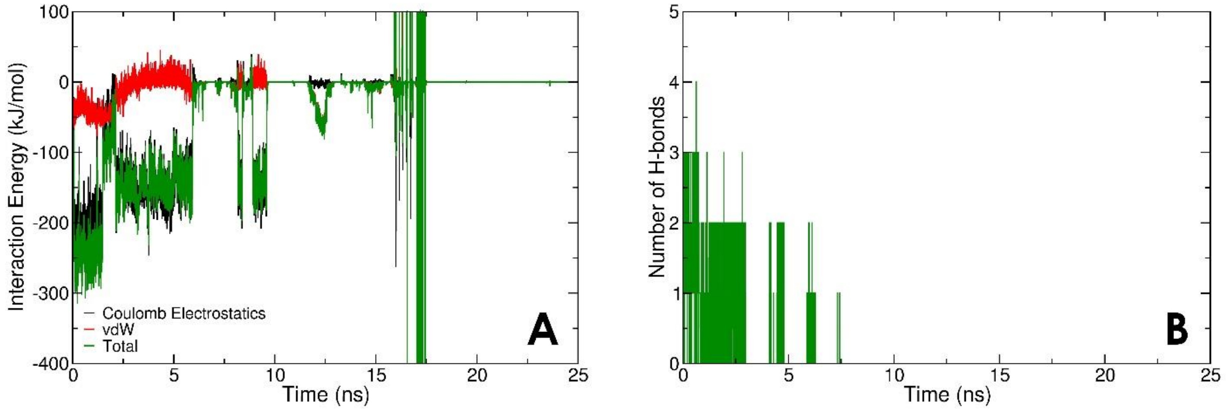

Figure S4. (A) The interaction energy and (B) number of hydrogen bonds between LPA and gelsolin(1-3)-actin in the first 25 ns of Dock 1.

S5. The Root Mean Square Deviation (RMSD) of Lysophosphatidic Acid

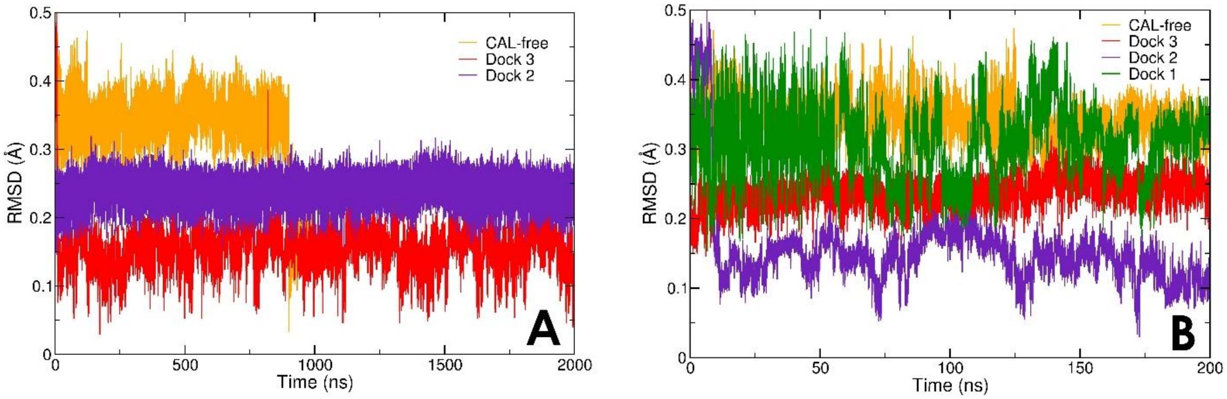

Figure S5. (A) The RMSD of backbone atoms of LPA, excluding Dock 1 as LPA leaves the binding pocket. **(B)** The RMSD of backbone atoms of LPA for Docks 1, 2, 3, and CAL-free in the first 200 ns.

S6-7. The Root Mean Square Fluctuation (RMSF) of Gelsolin(1-3) and Actin

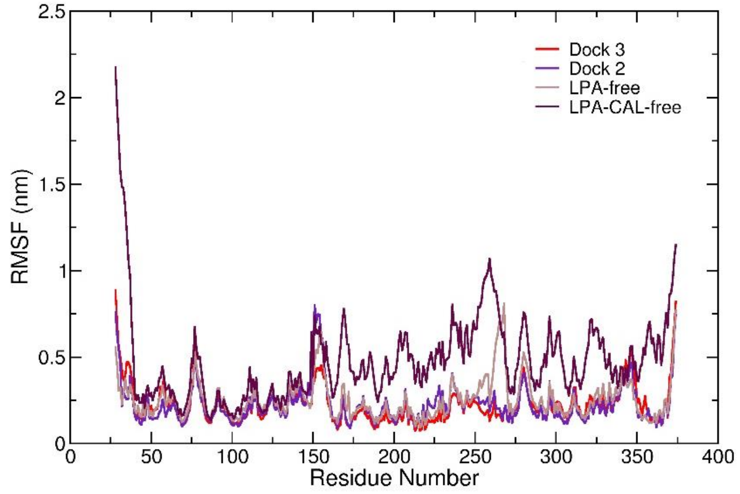

Figure S6. The root mean square fluctuation (RMSF) as a function of residue numbers of gelsolin 1-3 protein.

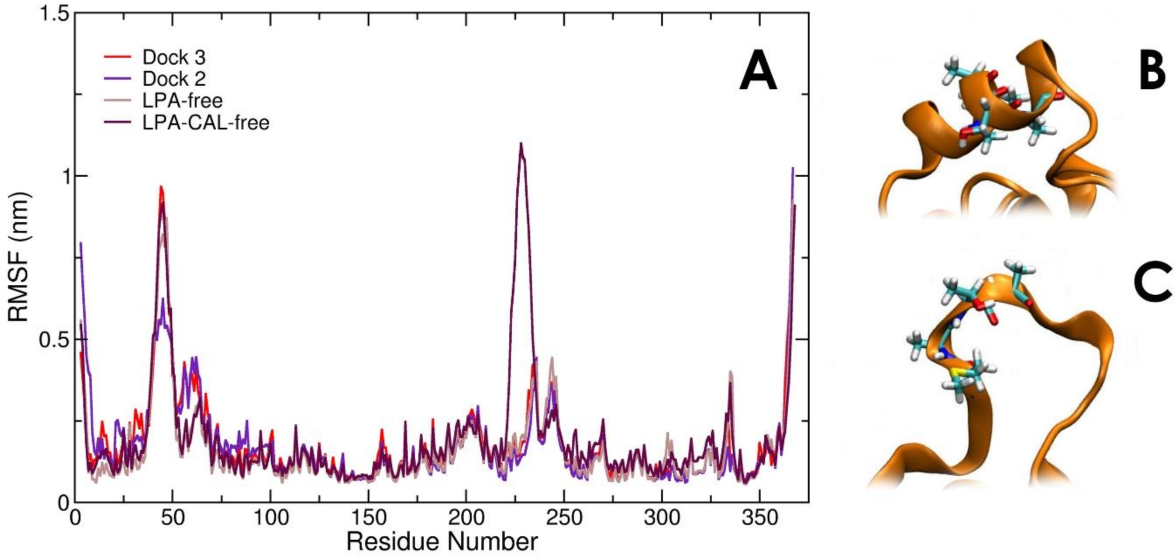

Figure S7. (A) The RMSF of actin. Actin shows the most structural fluctuation in the LPA-CAL-free system, where residues 227-230 have the most fluctuation as the alpha helix unfolds into a coil. (B) LPA-CAL-free residues 227-230 at 0 μs. (C) LPA-CAL-free residues 227-230 at 2 μs.

S8. Interaction Energy between Gelsolin(1-3) and Actin

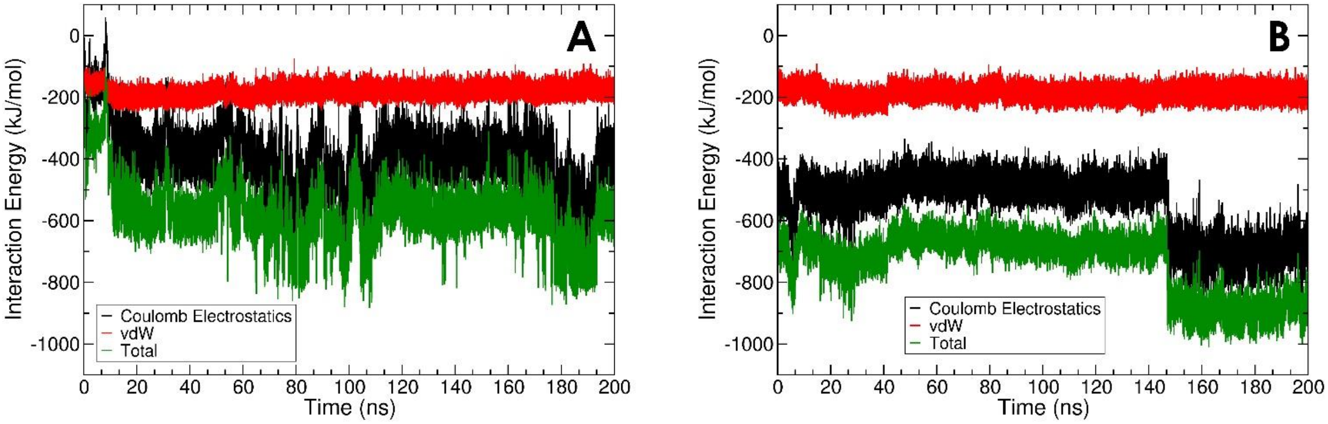

Figure S8. Interaction energy between gelsolin(1-3) and actin for the first 200 ns of (A) Dock 2 and (B) Dock 3.

S9-10. Secondary Structure and Conformational Energy of the Gelsolin(1-3)-Actin Complex

Ca^{2+} #2 ion leaves the binding pocket in the Dock 3 simulation ~1181 ns because of the protein complex's structural changes. As **Table S1** shows, the H-bond occupancy before and after the loss of Ca^{2+} #2 (~1181 ns) indicates no strong H-bond occupancy following ~1181 ns. As the LPA-free system's calcium ions remain bound to the protein during simulation time, this suggests that LPA binding in the Dock 3 system induces conformational changes that cause Ca^{2+} #2 to become less favourably coordinated, causing it to leave the binding pocket and subsequently weaken the protein's structural stability. In fact, the protein's structure experiences more variations (**Figure S9**) and less favourable conformational energy (**Figure S10**) in the second half of the simulation, when Ca^{2+} #2 is no longer bound to the complex.

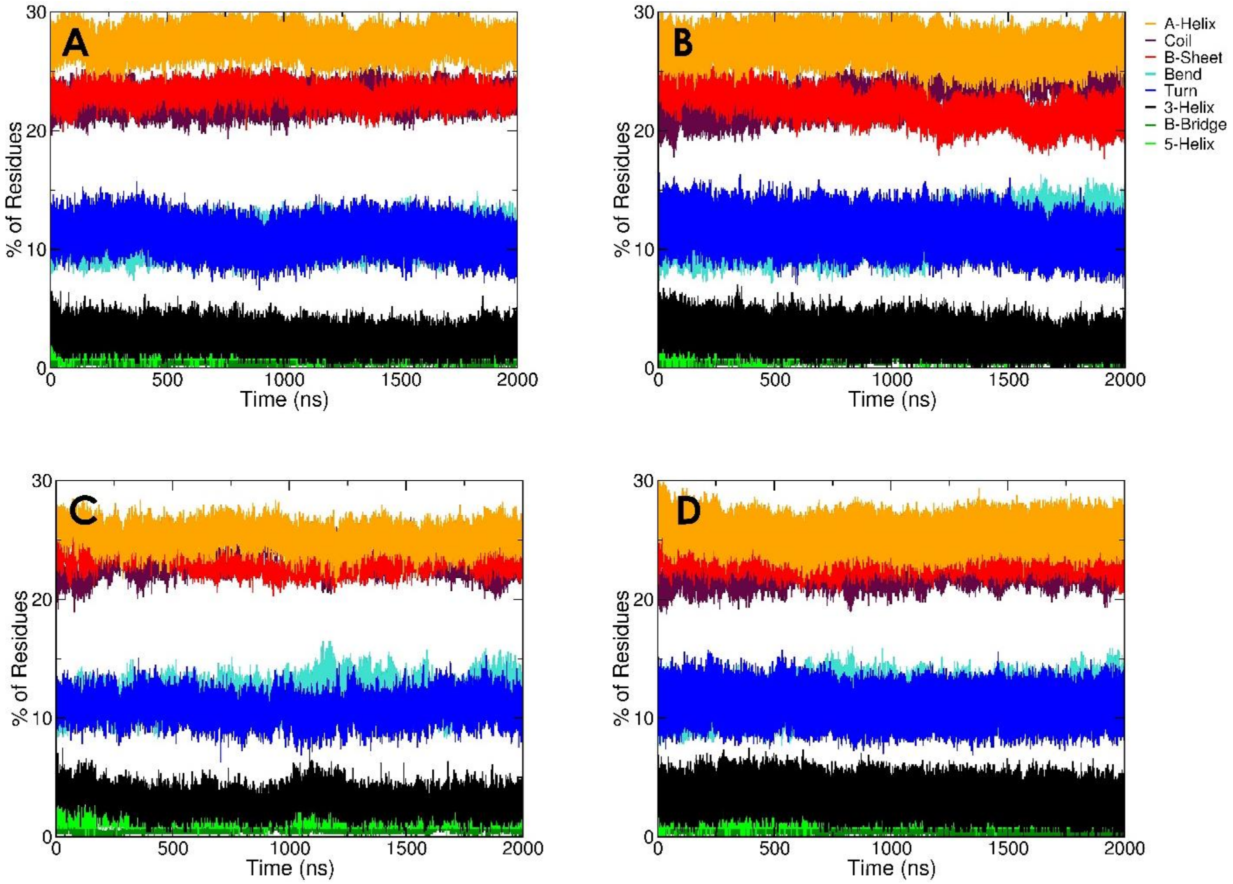

Figure S9. Changes in the secondary structures of gelsolin(1-3)-actin across simulation time for (A) Dock 2, (B) Dock 3, (C) LPA-CAL-free, and (D) LPA-free.

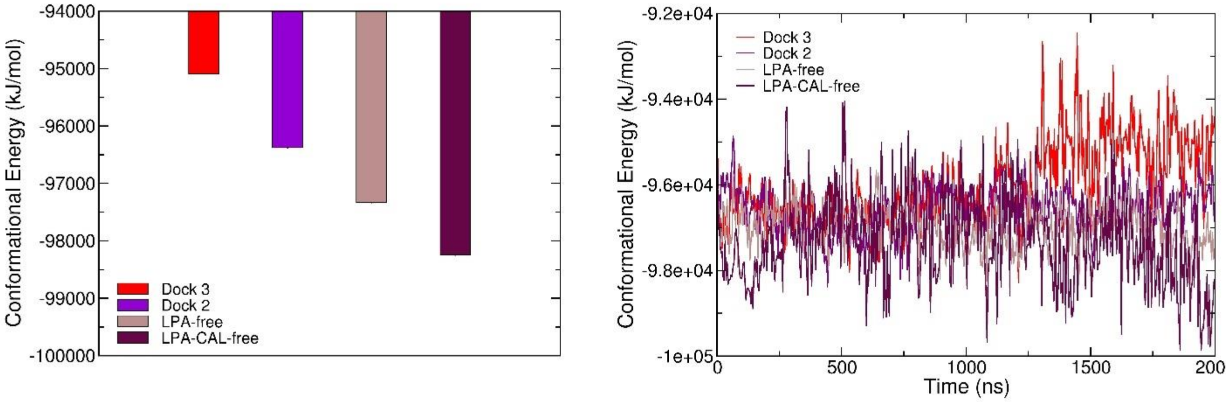

Figure S10. The conformational energy of gelsolin(1-3)-actin with normalized standard deviation for the bar graph. The standard error of the mean was calculated by block averaging [64].

With Ca^{2+} #2 unbound from gelsolin(1-3)-actin in Dock 3, strong hydrogen-bond occupancies are no longer present between gelsolin(1-3) and actin (**Tables S1 and S5**). The strongest H-bond occupancies before 1.18 μs become about 20-62% weaker following the loss of Ca^{2+} #2. In comparison to Dock 2, which maintains calcium ion binding with the protein complex throughout the simulation, strong hydrogen-bond occupancies are present between gelsolin(1-3) and actin after 1.18 μs (**Tables S4 and S5**). Dock 2 maintains the strongest hydrogen-bond occupancies in the protein complex relative to Dock 3, LPA-free, and LPA-CAL-free throughout the 2 μs dynamics (**Table S7**).

Interestingly, H-bond occupancy between the protein complex and LPA become slightly stronger by approximately 1-12% with the loss of Ca^{2+} #2 in Dock 3 (**Table S2**), suggesting that structural changes of the complex may take place which facilitate stronger hydrogen bonding between gelsolin(1-3)-actin and LPA. Such structural changes lead to less H-bond pairs between gelsolin(1-3) and actin in Dock 3 relative to Dock 2 (**Table S9**). Although Dock 3 has three less hydrogen bond pairs between gelsolin(1-3)-actin and LPA compared to Dock 2 (**Table S10**), more strong H-bond occupancies are present between LPA and the protein complex in Dock 3 (**Tables S2 and S4**). The different strong H-bond pairs between LPA and gelsolin(1-3) (**Tables S6 and S8**), together with the greater number in strong H-bond occupancies between LPA and gelsolin(1-3)-actin, suggest that LPA's binding position has a stronger influence towards decoupling the protein complex in Dock 3.

Table S1. Dock 3's Strongest (>70%) H-bond Occupancies between Gelsolin(1-3) and Actin.

Donor*	Acceptor*	Before 1.18 μs, with bound Ca^{2+} #2 (%)	After 1.18 μs, with unbound Ca^{2+} #2 (%)
THR148-Side	GLN118-Side	83.94	66.60
ARG228-Side	GLY13-Main	82.39	31.05
GLN118-Main	GLU167-Side	76.28	34.87
ARG228-Side	SER33-Main	74.93	30.59

*H-bond donors and acceptors can be from either gelsolin 1-3 or actin.

Table S2. Dock 3's Strongest (>70%) H-bond Occupancies between Gelsolin(1-3)-Actin and LPA.

Donor	Acceptor	Before 1.18 μs, with bound Ca^{2+} #2 (%)	After 1.18 μs, with unbound Ca^{2+} #2 (%)
SER147-Side	LPA770-Side	97.43	98.25
SER147-Main	LPA770-Side	86.81	87.93
LYS150-Side	LPA770-Side	76.24	86.25

Table S3. Dock 2's strongest (>70%) H-bond Occupancies between Gelsolin 1-3 and Actin.

Donor	Acceptor	Before 1.18 μs (%)	After 1.18 μs (%)
LYS319-Side	GLU100-Side	93.92	95.50
GLU28-Main	GLU361-Side	39.90	91.96
ARG120-Side	GLY146-Main	80.78	79.94
ARG328-Side	GLU100-Side	79.38	12.92
THR148-Side	GLN118-Side	79.30	82.87
ARG95-Side	GLU224-Side	30.40	80.82
GLN95-Side	ALA144-Main	71.04	82.88
SER350-Side	ASP50-Side	62.75	70.36

Table S4. Dock 2's Strongest (>70%) H-bond Occupancies between Gelsolin(1-3)-Actin and LPA.

Donor	Acceptor	Before 1.18 μs (%)	After 1.18 μs (%)
ARG147-Side	LPA770-Side	98.24	99.18
ARG120-Side	LPA770-Side	77.43	84.66

Table S5. The Number of H-bond Pairs between Gelsolin(1-3) and Actin.

Model	# of H-bond pairs before 1.18 μs	# of strong H-bond pairs before 1.18 μs	# of H-bond pairs after 1.18 μs	# of strong H-bond pairs after 1.18 μs
Dock 2	934	5	527	7
Dock 3	879	4	594	0
LPA-free	627	2	422	1
LPA-CAL-free	911	2	460	7

Table S6. The Number of H-bond Pairs between Gelsolin(1-3)-Actin and LPA.

Model	# of H-bond pairs before 1.18 μs	# of strong H-bond pairs before 1.18 μs	# of H-bond pairs after 1.18 μs	# of strong H-bond pairs after 1.18 μs
Dock 2	68	2	55	2
Dock 3	66	3	55	3

Table S7. Strongest (>70%) H-bond Occupancies between Gelsolin(1-3) and Actin across 2 μs.

Model	Donor	Acceptor	Occupancy (%)
Dock 2	LYS319-Side	GLU100-Side	94.57
	THR148-Side	GLN118-Side	80.76
	ARG120-Side	GLY146-Main	80.44
	GLN95-Side	ALA144-Main	75.89
Dock 3	THR148-Side	GLN118-Side	76.84
LPA-free	LYS150-Main	GLY23-Main	75.85
LPA-CAL-free	SER350-Side	ASP50-Side	85.66
	ARG221-Side	ASP244-Side	79.67
	THR148-Side	GLN118-Side	77.44
	GLN118-Main	GLU167-Side	72.47
	LYS218-Side	ASP244-Side	70.37

Table S8. Strongest (>70%) H-bond Occupancies between Gelsolin(1-3)-Actin and LPA across 2 μs.

Model	Donor	Acceptor	Occupancy (%)
Dock 2	ARG147-Side	LPA770-Side	98.62
	ARG120-Side	LPA770-Side	80.39
Dock 3	SER147-Side	LPA770-Side	97.77
	SER147-Main	LPA770-Side	87.27
	LYS150-Side	LPA770-Side	80.34

Table S9. The Number of H-bond Pairs between Gelsolin(1-3) and Actin across 2 μs.

Model	# of H-bond pairs	# of strong H-bond pairs (occupancy >70%)
Dock 2	1076	4
Dock 3	1029	1
LPA-free	708	1
LPA-CAL-free	1059	5

Table S10. The Number of H-bond Pairs between Gelsolin(1-3)-Actin and LPA across 2 μs.

Model	# of H-bond pairs	# of strong H-bond pairs (occupancy >70%)
Dock 2	72	2
Dock 3	69	3

Table S11. The Pearson coefficients between the interaction energies of gelsolin(1-3)-actin and the binding energies of gelsolin(1-3)-actin with LPA.

Model	Pearson Coefficients
Dock 2	0.075
Dock 3	0.037

Table S12. Residue-wise binding energies of Dock 3 during early and later stage dynamics.

Species	Before 148ns (kJ/mol)	1800 – 2000 ns (kJ/mol)
Gly23	-0.49	-0.54
Asp24	27.60	30.79
Lys150	-37.66	-67.13
Ca^{2+} #3	-436.39	-439.07
LPA	116.33	82.76

Supplementary References

38. Shelley, J. C.; Cholleti, A.; Frye, L. L.; Greenwood, J. R.; Timlin, M. R.; Uchimaya, M., Epik: a software program for pK(a) prediction and protonation state generation for drug-like molecules. *J Comput Aided Mol Des* **2007,** *21* (12), 681-91.

64. Grossfield, A.; Zuckerman, D.M. Quantifying uncertainty and sampling quality in biomolecular simulations. *Annu. Rep. Comput. Chem.* **2009,** *5,* 23–48.
